# Combined Open Surgical and Endoscopic Approach for Management of a Meningoencephalocele After Iatrogenic Perforation of the Anterior Skull Base in a Young Infant

**DOI:** 10.7759/cureus.24797

**Published:** 2022-05-07

**Authors:** Petra A Mercea, Anselm J Gadenstaetter, Christian Matula, Christoph Arnoldner

**Affiliations:** 1 Neurosurgery, Medical University of Vienna, Vienna, AUT; 2 Otolaryngology-Head and Neck Surgery, Medical University of Vienna, Vienna, AUT

**Keywords:** meningoencephalocele, iatrogenic perforation, open neurosurgical approach, endonasal endoscopy, preterm infant

## Abstract

Traumatic iatrogenic perforation of the anterior skull base is a rare complication following endonasal intubation in preterm infants. Subsequent meningoencephaloceles with concomitant cerebrospinal fluid (CSF) fistulas bear the risk of severe complications, therefore early diagnosis and closure of the skull defect are crucial. However, there is no consensus on the management of such cases of meningoencephaloceles. This case report presents a sophisticated approach of open brain surgery in combination with endonasal endoscopy. A 15-month-old girl presented with a meningoencephalocele and a CSF fistula due to iatrogenic perforation of the left anterior skull base during attempted endonasal intubation after birth. Difficult nasal breathing and an increasing diameter of the skull base defect on imaging controls indicated surgical management. Close multidisciplinary collaboration was essential for diagnosis and decision upon treatment. Open neurosurgical resection and CSF fistula closure combined with endonasal endoscopic removal of the excised meningoencephalocele was performed. Our case report shows that this combined open surgical and endonasal endoscopic approach is a safe procedure in favor of the postoperative outcome and follow-up of the patient.

## Introduction

Ethmoidal meningoencephaloceles are described as a herniation of meninges and brain tissue through a defect in the cribriform plate into the ethmoidal sinuses [1,2]. This can be either a congenital condition, or caused by trauma, or occurring spontaneously. However, in general, meningoencephaloceles are rare [2]. The exact incidence of skull base meningoencephaloceles is unknown and varies greatly between Southeast Asian countries (one in 5000-6000 births), the Middle East, and Western countries (one in 35,000-40,000 births) [2]. Ethmoidal meningoencephaloceles contribute to only 8% of the anterior skull base meningoencephaloceles [3]. Moreover, there exist only a few reports and case series of traumatic/iatrogenic anterior skull base meningoencephaloceles in the current literature [4-8]. In infants, especially preterm babies treated for respiratory distress syndrome, traumatic anterior skull base defects can occur as a result of failed endotracheal intubation through the nasal pathway. Additionally, a few case reports also describe an intracranial deviation of the endotracheal tube causing extensive cerebral trauma [5-7]. These anterior skull base defects can enable extracranial herniation of the meninges, brain tissue, and cerebrospinal fluid (CSF), resulting in the formation of a nasal meningoencephalocele [6,7,9]. Patients often present with rhinorrhea suggestive of a CSF fistula and nasal obstruction, and inadequate management can result in severe complications such as meningitis and seizures [2]. Furthermore, the meningoencephalocele can negatively affect the development of the craniofacial skeleton in infants [9]. The presence of a CSF leak already dictates the urgency of early treatment of the nasal mass and repair of the anterior skull base defect. The mainstay of the surgical management of meningoencephaloceles in pediatric patients is the open coronal approach via craniotomy and pericranial flap [8,9]. However, the endoscopic endonasal repair of meningoencephaloceles and CSF fistulas has gained more and more attention as it overcomes the limits of conventional intra- and extra-cranial skull base surgery [8-10]. In the presented case report, we describe for the first time a combined approach performed by neurosurgeons and otorhinolaryngologists via a direct open and endoscopic approach, for the rare complication of a meningoencephalocele due to endonasal endotracheal intubation in a preterm-born infant.

## Case presentation

**Figure 1:**
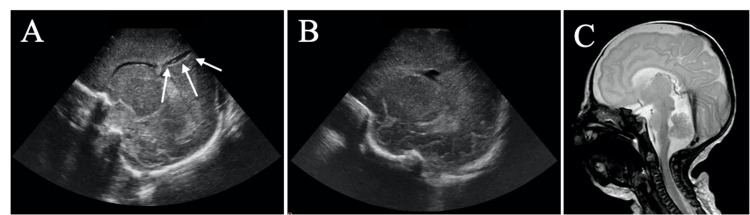
(A) Cerebral sonography of the newborn after difficult endotracheal intubation through the left nostril displaying a linear traumatic defect (white arrows) caused by the malpositioned nasotracheal tube. (B) Cerebral sonography and (C) MRI control with sagittal T2-weighted sequences after approximately three months show closure of the skull base defect. MRI: magnetic resonance imaging.

A 15-month-old girl was referred for evaluation and treatment of an ethmoidal meningoencephalocele to our university Department of Otorhinolaryngology at Vienna General Hospital. The patient was prematurely born (gestational week 24+3) and presented with poor vital signs and an absence of spontaneous respiration. Therefore, mechanical ventilation was indicated. After the first placement of the endotracheal tube through the right nostril failed, the tube was removed and placed through the left nostril. After initial light resistance, smooth protrusion of the tube was possible. However, it was not visible when inspecting the oropharynx. Serosanguineous fluid leaked out and the endotracheal tube was immediately withdrawn. Thereafter, a second attempt at intubation through the right nostril was finally successful. Early cerebral sonography displayed a linear defect of the brain parenchyma from the anterior skull base to the left parietooccipital cortex. This is consistent with a possible intracranial deviation of the nasotracheal tube. However, intracerebral hemorrhage was absent (Figure 1A). Although a pre-existing skull base defect prior to nasal intubation cannot be ruled out with certainty, we assume this to be an iatrogenic origin due to the history of failed intubation and the unlikely event of direct tube penetration through a pre-existing defect. In the further course, according to follow-up cerebral sonography the skull base defect appeared to have closed spontaneously (Figure 1B, 1C).

**Figure 2:**
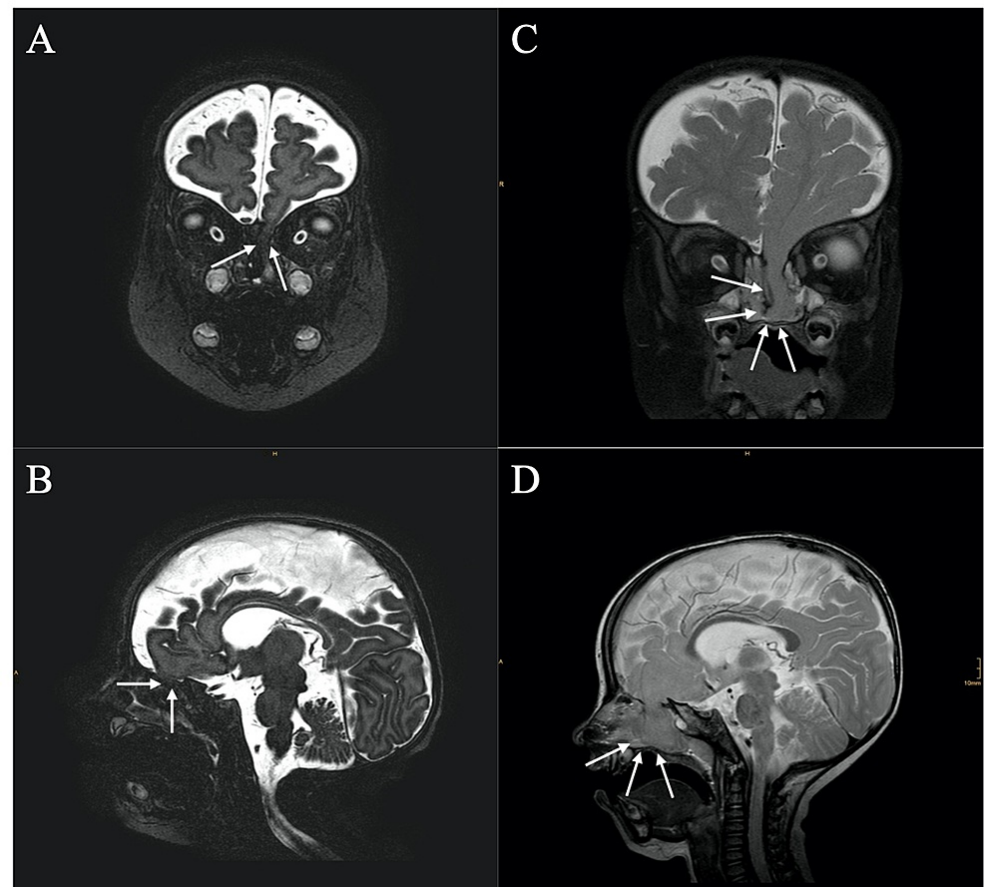
Strict MRI controls with coronal and sagittal T2-weighted sequences (A) and (B) approximately two months after birth, showing the bone defect of the anterior skull base with the concomitant meningoencephalocele (white arrows), (C) and (D) approximately six months after birth, displaying the assumed increase of the diameter of the bone defect as well as an enlarged meningoencephalocele (white arrows). MRI: magnetic resonance imaging.

Within two months, the patient demonstrated rhinorrhea, highly suspicious of CSF leakage. A cranial magnetic resonance imaging (MRI) showed a bone defect (1.2 mm in diameter) of the left lamina cribrosa, and two months later, a frontoethmoidal meningoencephalocele was present. Two months later, the control MRI no longer displayed the CSF flow voids through the anterior skull base defect but demonstrated a frontoethmoidal meningoencephalocele that reaching into the left middle ethmoidal cell. Since the young patient was neurologically asymptomatic, a strict wait-and-watch strategy was initiated. However, the bone defect increased and the meningoencephalocele protruded into the left ethmoidal sinus (Figure 2). 

**Figure 3:**
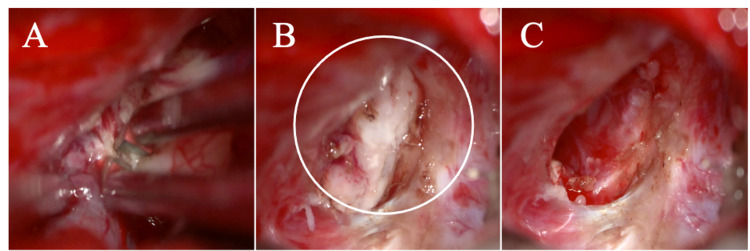
(A) Careful microsurgical detachment of intracranial frontal piece of the meningoencephalocele with bipolar caustic and suction device. (B) View through the operating microscope after removing the adhesions and protrusion of the frontal lobe, which was extending through the bone defect in the frontal skull base. The remaining intranasal part of the meningoencephalocele is visible (white circle). (C) After performing the endonasal endoscopic removal of the cele, the bone defect is easily detected from the intracranial perspective.

At admission to our clinic, the 15-month-old child presented neurologically stable, however, with obstructed airflow via the left nostril. Extensive multidisciplinary evaluation proposed combined surgical management including open craniotomy for disconnection of the meningoencephalocele and repair of the bone defect, as well as endonasal endoscopy for removal of the disconnected tissue. Due to the size of the bony skull base defect (Figure 3), the previous idea of purely endoscopic resection and defect closure was abandoned. 

Surgery was performed under general anesthesia at the Department of Neurosurgery with the assistance of neuronavigation (Medtronic Stealth Station S7). We performed a unilateral coronal incision and classic left lateral frontobasal osteoclastic trepanation. After the dura incision, the underlying arachnoidea was prepared and adhesions were carefully detached until the bone defect (1.5 cm in diameter) and the meningoencephalocele were visible. Due to the adhesions, the left frontal lobe seemed to be fused with the skull base. However, the meningoencephalocele could be disconnected at the fronto-basis with microscissors, suction, and cauterization. Solely endoscopic retraction or reposition through the bone defect could have had detrimental effects.

**Figure 4:**
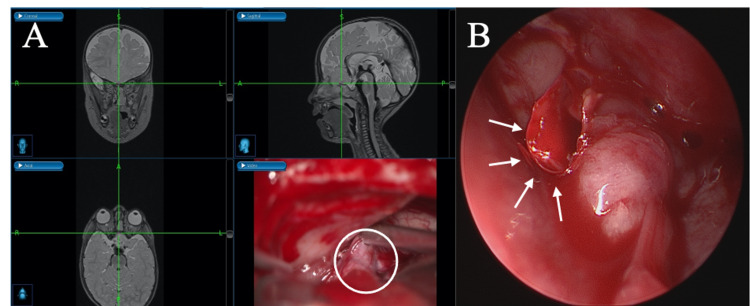
(A) Neuronavigation during intracranial microsurgical resection of the meningoencephalocele visualizes the almost exact localization of surgical equipment during the procedure. In the right bottom section, the upper part of the meningoencephalocele (white circle) is retracted by a suction device. (B) Endoscopic view of the endonasal pathway to retrieve the released meningoencephalocele (white circle) through the nose. The bone defect of the frontal cranial base is visible (white arrows).

**Figure 5:**
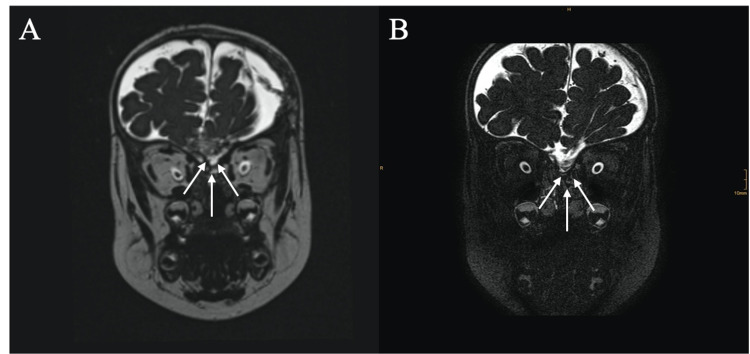
(A) A postoperative MRI control with a coronal T2-weighted sequence displays successful removal of the meningoencephalocele from the left ethmoidal sinus (white arrows). (B) At the six-month follow-up, the MRI control with coronal T2-weighted sequences shows complete closure of the skull base defect, thus a satisfactory result of the previously performed combined surgical approach for the treatment of the meningoencephalocele (white arrows).

The frontal lobe was easily repositioned, and the parenchyma was covered by Surgicel® (Johnson and Johnson Medical, Arlington, Texas). Next, by endonasal endoscopy, the disconnected meningoencephalocele was detached piece by piece from its surrounding adhesions (Figure 3, Figure 4) and retrieved via an endoscope (Figure 4). Complete removal was confirmed via an endoscope and neurosurgical microscope. In the end, via open surgery, the bone defect was covered by a TachoSil® Fibrin Sealant Patch (Takeda A/S, Corza Health, Inc., Linz, Austria) and a resorbable LactoSorb® plate (W.Lorenz Surgical, Jacksonville, Florida). A periosteum flap was performed for dura closure, and the operation site was closed in a multi-layer fashion. A lumbar drain was not required. The postoperative course was uneventful; the young patient was discharged on the seventh postoperative day. Postoperative MRI controls showed a satisfactory result. At the six-month follow-up, the girl presented with no neurological sequelae or behavioral anomalies. Additionally, the follow-up MRI displayed complete closure of the frontal skull base defect (Figure 5). A chronological, detailed course of events is listed in Table 1. Informed consent was provided by the mother for the publication of this case report.

**Table 1:** Timeline with information about historical, clinical, and interventional events. NICU: neonatal intensive care unit; RDS: respiratory distress syndrome; CSF: cerebrospinal fluid; MRI: magnetic resonance imaging.

Time of event	Clinical event	Intervention
Premature birth (gestational week 24+3)	No spontaneous respiration	3× Attempts of endonasal intubation
	Transfer to NICU ultrasound of cranium	Treatment of RDS linear brain defect from failed intubation; no hemorrhage wait and scan
Ninth week	Recovery from RDS persistent ductus arteriosus	Extubation ibuprofen therapy
Two months	Rhinorrhea	Beta-trace protein control, CSF MRI: anterior skull base defect
Four months	Spontaneous cessation of rhinorrhea	Control MRI: no skull base defect anymore but meningoencephalocele visible, wait-and-watch strategy
Thirteen months	No neurological deficits	Control MRI: increase of meningoencephalocele
Fifteen months	Status epilepticus	Anticonvulsants referral to our Department of Otorhinolaryngology, surgery planned in skull baseboard
Combined endonasal endoscopic and open surgical operation		Removal of meningoencephalocele and closure of skull base defect
Seventh postoperative day	Good clinical condition	Discharge from hospital
Eleventh postoperative day	Postoperative MRI	Satisfying postoperative result
Twenty-one months	Excellent condition six-month follow-up	Crawling, laughing, no behavioral anomalies, satisfactory control MRI

## Discussion

Meningoencephaloceles in newborn infants after endonasal endotracheal intubation have been reported in only a few cases in the literature [4-7,11]. Morphologically, meningoencephaloceles present as sac-like protrusions of meninges and brain tissue herniating through a skull base defect [7]. Nasopharyngeal meningoencephaloceles occupy the nasal cavity or the nasopharynx as a common feature [4,12]. Accordingly, the meningoencephalocele of our patient filled the nasopharynx and obstructed the natural airway through the left nostril. Moreover, through the dural defect, which was caused by trauma and the protruding meningoencephalocele, CSF leakage occurred and, simultaneously, the skull base defect showed size progression. Indeed, CSF fistulas are often associated with meningoencephaloceles, bearing the risk of bacterial meningitis with serious morbidity [13]. Due to the lack of meningitis and the absence of neurological deficits, a strict wait-and-watch strategy until the girl reached a suitable age for surgical treatment was initiated. Surgical treatment of meningoencephaloceles consists of maximal excision of the lesion and simultaneous repair of the bone defect either by a cranial approach via standard bicoronal incision or endonasal endoscopy [14-16].

The open surgical approach is a mainstay of management for anterior meningoencephaloceles as it is a robust and straightforward technique. However, it is associated with severe comorbidities [4,17]. In comparison, endonasal endoscopy is an effective alternative with fewer complications [7,17]. Mohindra et al. suggest that endonasal endoscopy is even superior to open surgery due to reduced morbidity [10]. However, failure of endoscopy with a rise of morbidity cannot certainly be excluded. In their case report, Renaud et al. describe the relapse of symptoms (i.e., rhinorrhea) and the requirement of a second look surgery approximately two weeks after the initial endoscopic intervention [7]. Therefore, after extensive discussion and evaluation of the benefits and risks for the patient, we decided upon the performance of a combined open and endonasal endoscopic approach. We believed that in this particular patient, regarding comorbidities and the extent and growth tendencies of the meningoencephalocele, the combined approach was the most suitable method to ensure the safe removal of the meningoencephalocele and definite closure of the skull base defect. We aimed to merge the advantages of both surgical approaches to minimize the risk for postoperative complications and revision surgeries. 

Congenital ethmoidal meningoencephaloceles and concomitant CSF fistulas are rare. However, both approaches, endoscopic as well as open surgery, have been described in the literature [18]. Nevertheless, we acknowledge that the combined surgical approach can only suit strictly selected patients and should always be discussed in specialized board meetings. However, to our knowledge, this is the first case report presenting the combined approach for the excision of an iatrogenic meningoencephalocele and repair of a CSF fistula in a young infant. Due to the unilateral coronal approach, retraction of the frontal lobes was minimized, and tight closure of the skull base defect was facilitated. The removal of the cele was performed via endoscopy, enabling a careful method appropriate for the young patient. One could consider a combined surgical approach as a time-consuming procedure associated with a higher risk of complications. However, in the postoperative follow-up, our patient presented in excellent condition without new neurological deficits.

## Conclusions

This is the first description of the repair of an iatrogenic anterior skull base defect and removal of an ethmoidal meningoencephalocele by a combined open surgical and endonasal endoscopic approach in a young infant. The postoperative outcome and clinical presentation at follow-up show complete removal of the meningoencephalocele and definite closure of the anterior skull base defect. We suggest the combined approach as a safe surgical procedure for closing skull base defects in young patients with complex meningoencephaloceles. However, we acknowledge that this approach can only suit strictly selected patients. Therefore, we recommend the critical discussion of cases and indications in specialized board meetings. Nevertheless, we regard the description of this combined surgical approach as helpful to aid decision-making in possible future pediatric patients with similar anterior skull base defects and ethmoidal meningoencephaloceles.
